# A fisheye viewer for microarray-based gene expression data

**DOI:** 10.1186/1471-2105-7-452

**Published:** 2006-10-13

**Authors:** Min Wu, Cheng Thao, Xiangming Mu, Ethan V Munson

**Affiliations:** 1 University of Wisconsin Milwaukee, Department of Health Sciences, 2400 E. Hartford Ave., Milwaukee, Wisconsin 53211, USA; 2 University of Wisconsin Milwaukee, Department of Computer Science, 3200 N. Cramer St., Milwaukee, Wisconsin 53211, USA; 3 University of Wisconsin – Milwaukee School of Information Studies, 3210 N Maryland Ave Milwaukee, WI 53211, USA

## Abstract

**Background:**

Microarray has been widely used to measure the relative amounts of every mRNA transcript from the genome in a single scan. Biologists have been accustomed to reading their experimental data directly from tables. However, microarray data are quite large and are stored in a series of files in a machine-readable format, so direct reading of the full data set is not feasible. The challenge is to design a user interface that allows biologists to usefully view large tables of raw microarray-based gene expression data. This paper presents one such interface – an electronic table (E-table) that uses fisheye distortion technology.

**Results:**

The Fisheye Viewer for microarray-based gene expression data has been successfully developed to view MIAME data stored in the MAGE-ML format. The viewer can be downloaded from the project web site . The fisheye viewer was implemented in Java so that it could run on multiple platforms. We implemented the E-table by adapting JTable, a default table implementation in the Java Swing user interface library. Fisheye views use variable magnification to balance magnification for easy viewing and compression for maximizing the amount of data on the screen.

**Conclusion:**

This Fisheye Viewer is a lightweight but useful tool for biologists to quickly overview the raw microarray-based gene expression data in an E-table.

## Background

The massively parallel research technique called *microarray *was developed ([1,2]) to take advantage of the unprecedented amount of information available about an organism's genetic makeup. Microarray enables researchers to measure the relative amounts of every mRNA transcript from the genome in a single scan, thus increasing the number of data points from an experiment by several thousand folds. Biological researchers, who have been used to studying a small number of genes thoroughly over a period of years, must now use new concepts and methods to store and analyze their experimental results. Because of variations in microarray technology and the immaturity of the field, the computation of gene expression level and usage of semantics varies between platforms. Minimum Information About a Microarray Experiment (***MIAME***) [3] was proposed in 2001 as a uniform standard for recording and reporting microarray gene expression data. MIAME has been widely adopted because it eases the interpretation of expression data and the independent verification of experimental results. An XML-based data format, Microarray-based Gene Expression – Markup Language (***MAGE-ML***), was developed to facilitate the exchange of MIAME data [4]. The MAGE-ML is the XML representation of MAGE-OM, which is an object model. The MAGE-OM contains 132 classes grouped into 17 packages. For example, Experiment is a package of MAGE-OM to describe the experiment goals and design; BioAssayData package stores gene-expression data; BioMaterial package describes biological materials used and description of their creation; and DesignElement package contains a mapping of features.

Biologists have been accustomed to reading their experimental data directly from tables. However, microarray data are quite large and are stored in a series of files in a machine-readable format, so direct reading of the full data set is not feasible. Even though a large amount of gene expression data can be integrated into tables, it is still difficult to browse. Incoming integrated bioinformatics systems will support simultaneous query on both knowledge databases and microarray data simultaneously. For example, FlyMine for *Drosophila melanogaster *is a data warehouse that integrates several genomic and proteomic data sets in one place and its website allows users to build arbitrary complex queries across all data [5]. But even when they have sophisticated tools like FlyMine, biologists will still want to preview the raw gene expression data in order to construct appropriate queries in the integrated database system. For example, a biologist is looking for the same gene expression profile from a microarray experiment. If the biologist can browse the raw data in the microarray, such as "ratio of means (ROM)" values, he or she can have a preliminary idea of the experimental data, such as the range of values. The biologist can efficiently use other computer systems to construct queries or confirm the accuracy of the query results.

The challenge is to design a user interface that allows biologists to usefully view large tables of raw microarray-based gene expression data. This paper presents one such interface – an electronic table (E-table) that uses fisheye distortion technology. Fisheye views have been widely used to deliver large amounts of data in limited screen space. Their use is motivated by the observation that, at any one time, users are only focusing on a small part of the data. Fisheye views use variable magnification: the data on which the user is focusing is large, neighboring data is smaller, and distant data is very small. The Fisheye technique was initially called "bifocal displays" [6]. As this "degree-of-interest" approach developed, it came to be called "fisheye views" [7] by analogy with the optical effect seen in photographs taken using fisheye lenses that have very short focal lengths. Table lens [8] is a "focus+context" based fisheye technology that works on tabular information to display of crucial label information and multiple distal focal areas. Graphical information is also integrated in the display of large tables using visualization technology [9]. Visualization help improve the presentation of tabular data because humans are good at spot patterns and features in well-designed graphical rendering of collection of values. In fact, the combination of fisheye and visual graphical technologies can reduce navigation time when viewing a large tabular data collection[8]. In this project, an E-table for MAGE data was successfully implemented as a Java application.

## Implementation

The Fisheye Viewer was implemented in Java so that it could run on multiple platforms. It uses the MAGE-ML Software Toolkit (MAGE-ML stk) [10] to read MAGE-ML [11,12] files. The MAGE-ML stk is itself based on the Xerces XML parser [13]. The majority of the viewer's interfaces were built using Netbeans Mantissa [14]. Rather than reinventing the wheel, we implemented the E-table by adapting JTable, a default table implementation in the Java Swing user interface library. This extended table class is called FishEyeTable and contains methods to provide focus. The variation of row height and font sizes is handled by the FishEyeTableCellRenderer class, which extends the DefaultTableCellRenderer class of the Java Swing library.

Fisheye views use variable magnification to balance magnification for easy viewing and compression for maximizing the amount of data on the screen. In our Fisheye Viewer, the user can click on any row to bring the focus to that row. The focus row is shown larger than all other rows and its text is larger and in bold face. The height and font size of other rows is determined by their distance from the focus row, with row height and font size becoming progressively smaller as the distance from the focus row grows. The height of the focus row is determined when the focus method of the FishEyeTable class is called by a selection listener. Once the row height is determined, the cells of the row are rendered using a corresponding font size by the FishEyeTableCellRenderer class. The heights and text fonts of the neighboring rows are controlled by a ListListener that observes changes in the user's selection. A separate listener is required for the neighboring rows because of the protocol used by the classes of the JTable package in Swing. Finally, because the tables are too large to show in their entirety on a single screen, a scroll pane allows the user to scroll the table up or down to see the hidden rows.

For gene expression data tables, it is useful to have both column and row headings, but JTable and the default table model only support column headings. In a non-fisheye table, a list could be used as the row header. However, list item heights are fixed, so there would be no way to vary the heights of the rows based on the focus. So instead, we used a second E-table just for the row headers. Then, we extended the ListListener class to be able can notify multiple tables and attached it to the FishEyeTable object. So, when a row is focused in the data table, the row header is also notified which to set its focus on the corresponding row header.

## Results

**Figure 1:**
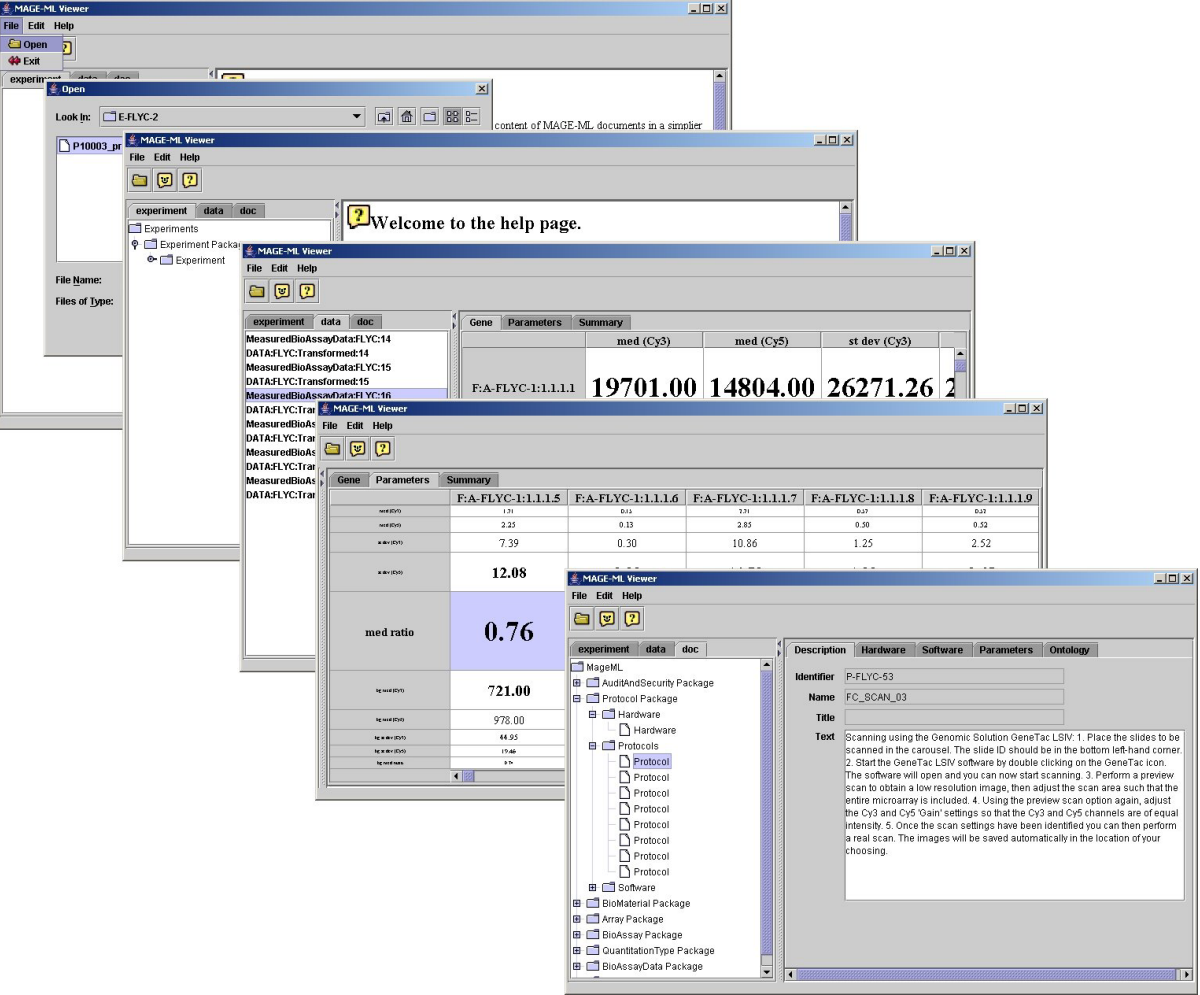
User interfaces of Fisheye Viewer.

The Fisheye Viewer for microarray-based gene expression data has been successfully developed to view MIAME data stored in the MAGE-ML format. The viewer can be downloaded from the project web site [15]. MIAME has six major components as follows. (1) *Experimental design*: the set of hybridization experiments as a whole; (2) *array design*: each array used and each element (spot, feature) on the array; (3) *samples*: samples used, extract preparation and labeling; (4) *hybridizations*: procedures and parameters; (5) *measurements*: images, quantification and specifications; and (6) *normalization controls*: types, values, and specifications. The viewer can read MAGE-ML files and map the structure of the document to a tree structure for navigation. The Fisheye Viewer was tested on microarray experiment data in MAGE-ML format. The viewer allows users to view each component of the microarray data (see Figure 1).

**Figure 2:**
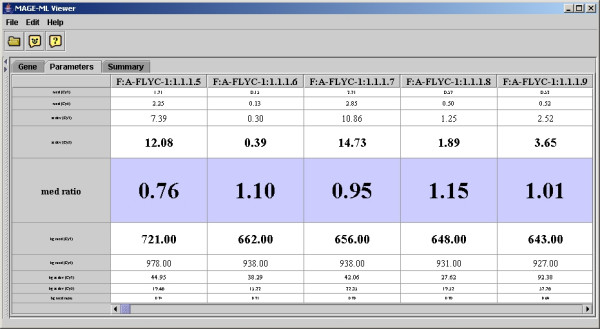
Focus on a parameter of interests.

**Figure 3:**
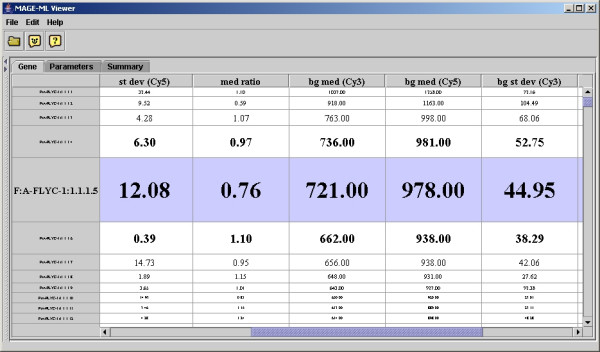
Focus on a specific gene of interests.

**Figure 4:**
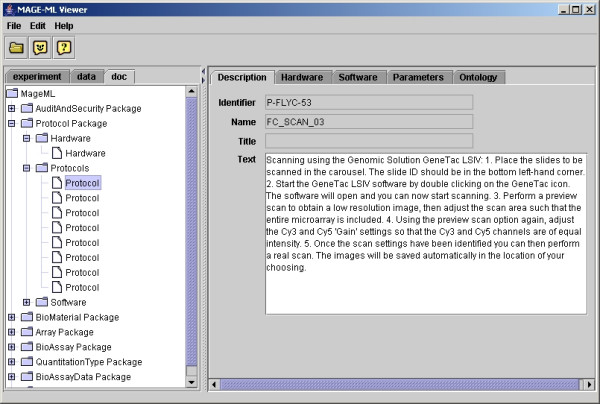
User interfaces for viewing protocol information.

The E-table in the application allows the users to navigate through gene expression data values. The tool allows users to view the gene table with the row representing parameters and the columns representing genes, so users can compare a parameter across all the genes. If the user clicks the row for "median ratio", the viewer brings the focus to that row (see Figure 2). The user can clearly view median ratio values and see the range of values for this parameter. If users identify a gene of interest, such as one with the largest ratio value, the user can use the application to view the gene with whatever MAGE-OM DesignElement is actually being displayed (Feature, Reporter, CompositeSequence, etc.) (See Figure 3). The viewer also allows the user to see other MIAME data components, such as protocols and contacts (see Figure 4). The user can use the tree on the left panel to select an element of interest.

A pilot user study was conducted to assess the user usability of this software. We used the Questionnaire for User Interface Satisfaction (QUIS) [16] as the evaluation instrument and recruited five volunteers to participate the study. The participants were three faculty members, one post-doctoral fellow and one graduate student from three laboratories in the biological sciences department at UWM. After participants used this tool at their offices, they completed the QUIS questionnaire. The users' evaluation are summarized and presented in a table (see Table 1).

**Table 1:** The result of a pilot user study

	User 1	User 2	User 3	User 4	User 5	Mean
Overall reaction	9.0	9.0	9.0	6.0	8.0	8.2
Screen	7.8	9.0	9.0	6.5	9.0	8.3
System information	8.8	9.0	9.0	8.0	9.0	8.8
Learning	9.0	9.0	9.0	8.3	6.2	8.3
System capabilities	9.0	9.0	9.0	6.8	9.0	8.6
Average	8.7	9.0	9.0	7.1	8.2	8.4

User feedback was quite positive, with a mean overall reaction score of 8.2 (on a scale from zero to nine). The QUIS scale with the highest score was the system information scale (8.8), which includes 1) use of terms throughout system, 2) terminology related to task, 3) prompts for input, 4) computer informs about its progress, and 5) error messages. User 2 and User 3 gave all nines, the highest scores to each item, while Users 1 and 5 gave many scores of 9. User 4, however, gave only 7.1 on average to the software and he also questioned the usefulness of the software. In particular, he indicated that he did not think this software organized information well on the screen (1 from 0–9 level) and did not designed for all levels of users (2 of 0–9 scale). User 4's overall reaction score was only 6.0. The inconsistency of the user responses in the pilot study indicated that further improvements to the software will be needed for it to gain wide acceptance. However, the enthusiastic response of some users indicates that the system shows real promise.

## Discussion

The design of the E-table is generic for large tabular data, so it could also be integrated into other biological data warehouses to preview the data before constructing complex queries or to confirm the results after the queries.

The sizes of MAGE-ML files vary greatly: some are only several megabytes while other can require hundreds of megabytes. If the user attempts to view a large MAGE-ML file will cause an out of memory error. This is the nature of MAGE-ML documents: they are simply huge. We have tested the application on a desktop PC with 512 MB of RAM running Windows XP and Linux, and on a Tablet PC with 512 MB of RAM running Windows XP Tablet PC edition. The sample MAGE-ML files used were around 1 – 3 MB in size.

## Conclusion

A new MAGE data viewer using fisheye distortion technique was successfully developed. The viewer can be used to view most types of data elements in the MAGE-ML format. This Fisheye Viewer is a lightweight but useful tool for biologists to quickly overview the raw microarray-based gene expression data in an E-table. The software package is made freely available for the scientific community via the project web site [15].

## Availability and requirements

Project name: Fisheye Viewer for Microarray-based Gene Expression

Project home page: 

Operating system(s): Platform independent

Programming language: Java

Other requirements: Java JRE 1.4, 512 MB of RAM or more

License: N/A

Any restrictions to use by non-academics: N/A

## Authors' contributions

MW identified the need for improving viewing interfaces for microarray-based biological data and guided the user interface design process. CT implemented the Fisheye Viewer application and its E-table component. XM designed and supervised the implementation of the fisheye algorithm. EVM revised the paper. All authors read and approved the final manuscript.
